# Enhancing corrosion-resistant alloy design through natural language processing and deep learning

**DOI:** 10.1126/sciadv.adg7992

**Published:** 2023-08-11

**Authors:** Kasturi Narasimha Sasidhar, Nima Hamidi Siboni, Jaber Rezaei Mianroodi, Michael Rohwerder, Jörg Neugebauer, Dierk Raabe

**Affiliations:** ^1^ Max-Planck-Institut für Eisenforschung GmbH, Max-Planck Straße-1, 40237 Düsseldorf, Germany.; ^2^ Ergodic Labs, Lohmühlenstraße 65, 12435 Berlin, Germany.

## Abstract

We propose strategies that couple natural language processing with deep learning to enhance machine capability for corrosion-resistant alloy design. First, accuracy of machine learning models for materials datasets is often limited by their inability to incorporate textual data. Manual extraction of numerical parameters from descriptions of alloy processing or experimental methodology inevitably leads to a reduction in information density. To overcome this, we have developed a fully automated natural language processing approach to transform textual data into a form compatible for feeding into a deep neural network. This approach has resulted in a pitting potential prediction accuracy substantially beyond state of the art. Second, we have implemented a deep learning model with a transformed-input feature space, consisting of a set of elemental physical/chemical property–based numerical descriptors of alloys replacing alloy compositions. This helped identification of those descriptors that are most critical toward enhancing their pitting potential. In particular, configurational entropy, atomic packing efficiency, local electronegativity differences, and atomic radii differences proved to be the most critical.

## Teaser

Deep learning frameworks utilizing experimental data enhance machine comprehension related to metallic corrosion.

## INTRODUCTION

Within only a few years, machine learning has revolutionized the way how we design advanced materials (*1*–*4*). Rapid progress has been made particularly due to the initiatives within the materials research community to systematize the protocols for collection and documentation of materials data in a manner amenable to analysis by machine learning. Large-scale initiatives such as the Materials Project (*5*), Citrination, and JARVIS (*6*) are noteworthy examples. Of all the many directions that have been pursued, few of the important ones are as follows: (i) property and performance prediction of materials in an engineering system with a given set of input parameters (including for instance, processing history, and service conditions) (*7*, *8*), (ii) discovery of new material compositions and processing routes for achieving application-oriented targets in terms of desired material properties (*9*, *10*), and (iii) image-based analysis methods for automating materials characterization (*11*). In addition, another direction that is beginning to gain importance, especially in view of developments in “explainable artificial intelligence (AI)” (*12*) is to derive mechanistic insights into pertinent materials properties/processes from machine learning–based methods, i.e., transitioning from correlations toward causality (*13*). Two categories of methods that enhance the explainability and interpretability of machine learning based models can be identified: (i) “model-agnostic methods,” which can be applied to any machine learning model regardless of the algorithm, and (ii) “model-specific methods,” which analyze specific internal components of a model and are thus specific to the particular algorithm (*12*, *14*). Examples of the first category include feature permutation (*15*), input perturbation analysis, and automated reasoning-based approaches (*14*), while methods such as layer-wise relevance propagation (*16*, *17*) and gradient-based sensitivity analysis (*18*) are model-specific approaches to analyze deep neural networks (DNNs).

Different aspects of metallic corrosion have been studied in recent years through one or more of the above-mentioned machine learning approaches, in the hope of identifying efficient means to tackle this menacing problem, contributing to annual economic losses of the order of 2.5 trillion USD (*19*). Prediction of high-temperature oxidation kinetics of alloys (*20*, *21*), atmospheric corrosion rates of steels (*22*, *23*), environmental corrosion in reinforced concrete structures (*24*), and identifying forms of corrosion/coating materials from images using convolutional neural networks (*25*, *26*) are examples in this regard. The effectiveness of the different machine learning algorithms over different types of datasets and problem statements of this category has been reviewed in (*27*). In the direction of alloy discovery, attempts have been made to create automated workflows making use of programmable scanning flow cells and additive manufacturing for rapid experimental data generation (*28*, *29*). Such high-throughput experimental methods coupled with machine learning have served to identify optimized alloy compositions, for example, within the Fe-Cr-Ni-Mn system for molten salt corrosion resistance (*28*) and an optimized Al-Ni-Ti alloy with high corrosion resistance (*29*). In the context of explainable AI, a recent work has used a gradient boosting–based feature ranking algorithm to identify the most critical parameters affecting the uniform corrosion rate of multiprincipal element alloys (*30*).

We have recently developed a DNN model for analyzing compositional and environmental contributions toward pitting resistance of several classes of corrosion-resistant alloys (*31*). In this introductory work, a rather simplistic DNN was trained to predict the pitting potential of an alloy of a known bulk composition in a given electrochemical test environment. The simplicity of the network lied in the fact that it was capable of accepting only numerical inputs consisting of alloy composition and environmental test parameters including test solution pH value, chloride ion concentration, and test temperature. However, the phenomenon of pitting in corrosion-resistant alloys is systemic in nature, i.e., it is dependent on the precise microstructural state of the material and the dynamics of the electrochemical tests performed, over and above the average alloy composition. Consequently, machine capability to predict the pitting potential of an alloy can only be enhanced by taking information pertaining to alloy-processing history (that governs its final microstructural state) and the experimental protocols followed during the electrochemical testing into account. In view of the fact that such information is routinely presented in the form of detailed text descriptions (for example, in literature references), making such information machine comprehensible is not straightforward. It can be achieved by a process of manual feature extraction, for instance, of information such as heat treatment temperature, time, electrochemical cell construction, etc. from the textual descriptions. However, such a manual process is not practicable due to multiple reasons: (i) It is not scalable to large datasets [especially with the trend moving toward creating large datasets through automated text mining (*32*, *33*)]; (ii) considering the heterogeneity in information in the context of electrochemical corrosion testing, it would be not possible to ascertain the most important features/information to be extracted from the text; (iii) information density could be substantially compromised. Therefore, a completely automated natural language processing approach (i.e., an approach that can transform textual data into a meaningful numerical data structure without human intervention), coupled with a deep learning model is essential to process such textual data and thus contribute to more accurate predictions on alloy corrosion behavior (*32*–*35*). Further, while it must be mentioned that optimizations carried out using the simplistic neural network model proved to be useful (*31*), the specific mechanisms by which different elements possibly enhance the pitting resistance of the alloys (in the spirit of explainable AI) could not be discerned. It must be said, therefore, that although the approach has allowed us to make an initial evaluation of its potential in general, substantial room for improvement exists in different directions.

In this work, we have attempted to enhance the capabilities of the deep learning framework to predict the pitting potential of corrosion-resistant alloys in two principal directions. First, we have implemented a model coupling an automated natural language processing methodology with a DNN to facilitate training on both numerical and textual data simultaneously. This has, as explained above, helped enrich the information density within the training data, resulting in a substantial improvement in its training and test accuracy. Second, we have made an attempt to train a simple DNN model with a transformed input feature space. In other words, the input composition of each alloy in the dataset is transformed into a set of “alloy descriptors” making use of different atomic, physical, and chemical properties of the constituent alloying elements. With the help of model training and optimization, fundamental alloy characteristics, independent of the elements involved, that could be most important for pitting resistance could be identified. We also demonstrate the capability of this approach of input feature space transformation to evaluate alloy systems that are completely absent in the training data.

## RESULTS AND DISCUSSION

### Process-aware DNN

#### Model architecture

The dataset on “electrochemical metrics for corrosion-resistant alloys” (*36*), as adapted for pitting potential in (*31*) (with a total of 769 records across five alloy classes) has been used in this work. The dataset consists of three categories of input features: (i) numerical features, including alloy composition, pH value, and chloride ion concentration of the test solution and test temperature; (ii) categorical features, including microstructure and material class; and (iii) textual features, including heat treatment, test method, comments, and scan rate. The textual input features documented in the dataset consist of excerpts from the respective published literature references, describing certain details of the alloy processing history (in the “heat treatment” and “comments” features), and experimental test methodology (in the “test method” and “scan rate” features) that could influence the pitting potential. Some of these textual data instances, as documented in the dataset are exemplarily shown in Table 1.

**Table 1.** Examples of the types and content of textual data instances in the dataset. The textual data instances are excerpts from different sections of the literature references from which the dataset was compiled. All four of the textual features are fed as input into the model for training.

Test method	Scan rate	Heat treatment	Comments
Potentiostatic polarization. The experimental procedure was to first polarize the electrode potentiostatically at the least noble potential within the passive region for at least 15 min to achieve steady-state passivity. The potential was then advanced in stages of 50 mV, allowing 5 min between each change. Initially, the current decreased or remained constant after each potential adjustment, but eventually, a steadily increasing current at some potential V_c_ indicated the onset of pitting (or of transpassivity). Steady-state values V_c_ were obtained subsequently by holding the potential for long times at a fixed value and then observing pitting or the lack of it under a low-power microscope. The lowest potential for which pitting could not be observed after a 10-hours or longer period of constant polarization was considered to be the steady-state value V_c_. Reproducibility was in the order of ±5 mV.	Steady state	The ingots were homogenized in A or He, usually at 1050°–1100°C for several hours. Ingots of Ni-Cr, Ni-Mo, and Cr-Fe were cold rolled to 0.25 cm, then annealed at 1000°–1050°C and water quenched. The Cr-Fe alloys containing more than 40% Cr were difficult to roll or swage; hence, electrodes were machined directly from the homogenized ingot. Ingots of the lower % Cr-Fe alloys and of the stainless steels were swaged to about 0.45-cm-diam rods, annealed at 1050°C and water quenched. All electrodes measured approximately 2 cm long and were either 0.4 cm diam or 0.5 cm wide by 0.2 cm thick.	The electrodes were abraded to a final 3/0 emery paper, and pickled usually in 15% HNO_3_, 5% HF at 80°C for 5 min to remove the cold worked surface.
The “scratch” technique was used for the determination of pitting potentials. The electrode was first polarized potentiostatically to some potential well below the expected pitting potential. The polarization current was allowed to stabilize and then the electrode surface was scratched with a sharp silicon-carbide crystal mounted on the end of a glass tube. The scratching of the electrode surface resulted in a sharp polarization current “blip.” The electrode potential was manually adjusted by 20-mV steps to more and more noble potential values, until the scratch failed to repassivate, a failure that was indicated by a gradual current rise after the electrode was scratched. The pit was allowed to develop for 10 min, and then the electrode was examined visually. Only those results were considered to be valid where pits actually developed at the scratched site.	20-mV steps by manual increment	Heat treated for 1 hour at 1500°F followed by water quenching.	Polished only through 600-grit SiC metallographic paper.

**Fig. 1.**
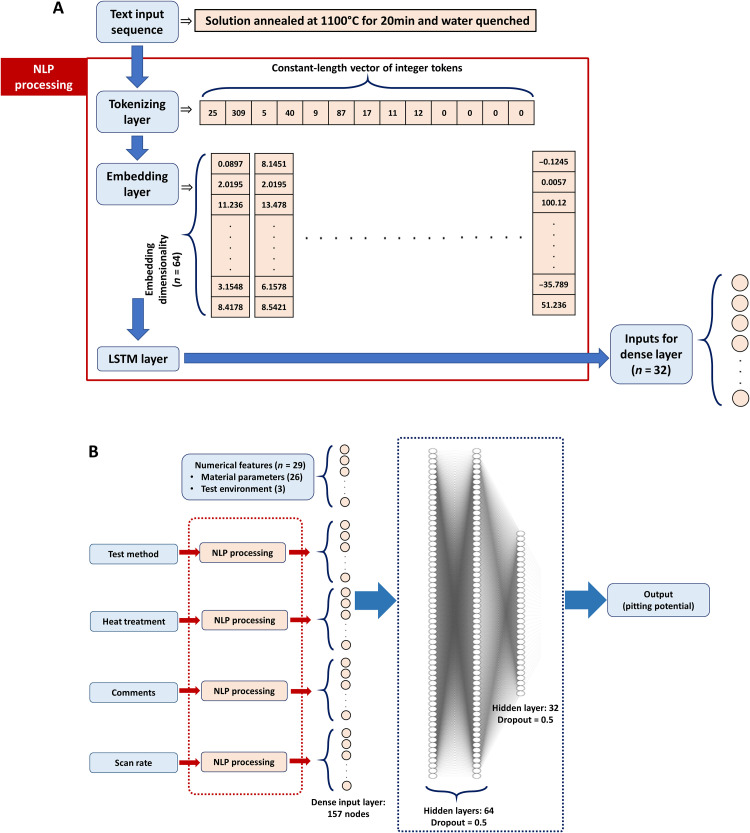
Process aware DNN model architecture. (**A**) Schematic representation of the entire process-aware DNN model. (**B**) Schematic illustration of the data processing workflow carried out within the natural language processing (NLP) module. LSTM, long-short–term memory.

For the purpose of serving as input data for training a DNN, the numerical inputs are taken as such, and the categorical inputs are transformed into numerical inputs by serial numbering [as in (*31*)]. In case of the textual input features, a natural language processing architecture (schematically illustrated with the help of an example in Fig. 1A) has been used for transforming them into an amenable form. The approach consists of three main stages, namely, tokenization of the vocabulary, word embedding (or word vectorization), and last, processing the sequence of embedded vectors corresponding to each phrase/sentence of a particular input instance through a recurrent neural network (RNN) layer. Word embedding combined with RNNs (to process data with a sequential nature) has emerged as a popular method for most text-related automation tasks including sentiment extraction, text completion, language translation, and smart human assistance (*37*–*40*). This has primarily been due to their ability to capture “context” in textual inputs, deal with words/phrases not encountered during training, learn grammatical constructs, and maintain a high degree of information density, as opposed to conventional NLP approaches. The method and architecture by which this is accomplished by these models is described below.

In the first step of tokenization, each unique word in the entire dataset is replaced by a unique integer token (a dictionary size of 10,000 was used as a safe upper bound for the current dataset). This would transform a given phrase/sentence into a vector of integers as shown in Fig. 1A. Since different phrases/sentences can have different sequence lengths, the vector of integer tokens is padded with a requisite number of zeroes at the end to obtain constant-length vectors of size equal to the maximum sequence length in the dataset (an upper bound of 200 has been used).

However, these integer tokens corresponding to the words do not carry any interpretable meaning. For instance, they cannot represent the semantic similarity between different words or part of speech of a given word. To achieve this, the next step of word embedding or, respectively, word vectorization is carried out. In this process, each integer token is converted into an *n*-dimensional vector of floats, where *n* is the embedding dimensionality (a value of *n* = 64 has been used in the current work) (https://www.tensorflow.org/api_docs/python/tf/keras/layers/Embedding). Weights for performing this operation are optimized during training of the model, i.e., it is a supervised embedding process, for the purpose of generating context-specific embedding vectors. Upon completion of training, the closeness of vectors corresponding to each word in the embedding space represents their semantic similarity. Thus, each vector of integer tokens in the input data is converted into a second rank tensor as shown in Fig. 1A.

In the final step, such a second-rank tensor consisting of embedded word vectors is processed through a RNN layer, to convert each tensor into a single vector (carrying 32 elements in this case) that can be fed as input to a fully connected dense layer. A long-short–term memory (LSTM) layer was used for this purpose. These layers have been found to be most efficient to handle sequential data (such as text, audio, and video) (*41*). With the help of introducing gate functions within the cell structure, these layers have the capability to identify long-term dependencies. In the current context, they would have the capability to identify key, related words in a given phrase/sentence, regardless of their position in the sequence of words (*41*). Thus, the most meaningful portion of the sentence would be supplied as input to subsequent layers of the model.

Last, all the numerical inputs processed through a normalization operation and all the output elements from the LSTM layer of each of the textual inputs are concatenated to form the input layer to a fully connected DNN model (Fig. 1B). Three subsequent fully connected hidden layers, with similar hyperparameters as optimized in (*31*) [i.e., 64, 64, and 32 nodes, respectively, equipped with a rectified linear unit (ReLU) activation function and dropout fraction of 0.5 at each layer] have been used before reaching the final output, i.e., the pitting potential. This entire architecture has been termed as the “process-aware DNN model,” custom-designed for predicting the pitting potential of an alloy composition with a given processing history and measured under a given set of test conditions.

#### Training and validation

**Fig. 2.**
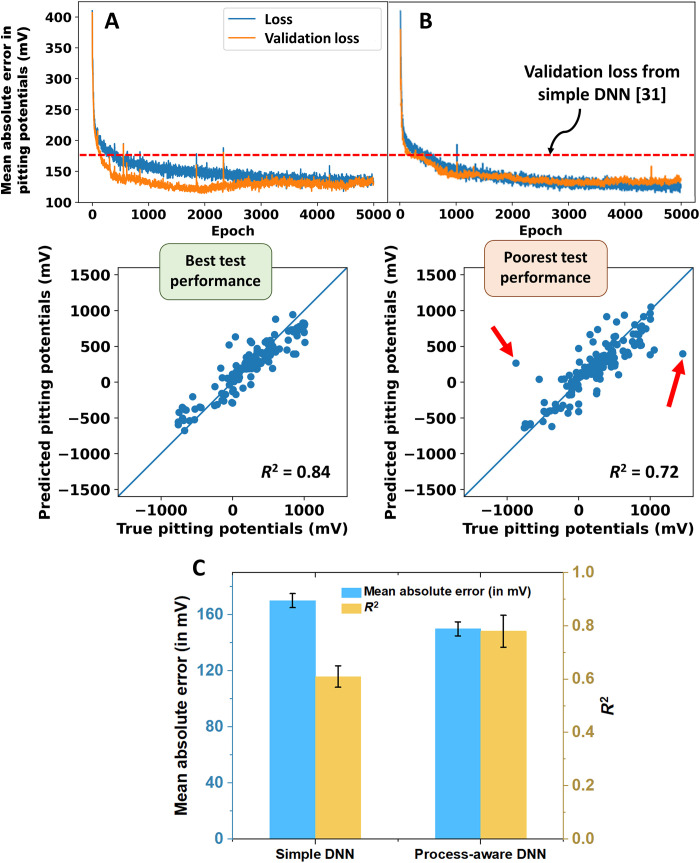
Model-training results for the process-aware DNN. Variation of the train and test accuracies (termed as the loss and validation loss, respectively) during the training history and predictions of the trained model over the test dataset at the end of training are shown. The average validation loss obtained after training of the simple DNN (*31*) has also been indicated. The variation in performance during different folds of the sixfold cross validation has been represented by showing the best (highest *R*^2^ coefficient and lowest validation loss at the end of training) and worst performances in (**A** and **B**), respectively. (**C**) Comparison of the model evaluation metrics for the simple and process aware DNNs.

Figure 2 shows representative results of the evolution of the mean absolute error values of network predictions on the training and test datasets during 5000 epochs of training history. The two sets of plots correspond to the best and worst performance during the sixfold cross-validation, respectively. The mean absolute error values on training and test datasets, also referred to as the loss and validation loss, respectively, can be seen to saturate in both cases at a minimum value close to 150 mV (Fig. 2), in comparison to the 170 mV obtained in case of the simple DNN (*31*), at the end of training. The difference in performance can be observed in the *R*^2^ coefficient being 0.72 and 0.84, respectively. After the sixfold cross-validation in training, an average validation loss value equal to 150 mV, and an average *R*^2^ coefficient of 0.78 ± 0.06 over test data predictions have been obtained. It can thus be seen that the present deep learning architecture taking the material processing history and details of test methodology into consideration outperforms the previous simplistic DNN (which also had a lower average *R*^2^ coefficient of 0.61 ± 0.04), when trained over the same dataset (*31*). The variation in test accuracies across different folds of the cross-validation is a consequence of specific outlier data points (marked by red arrows in the scatter plot in Fig. 2B). Analysis of the train-test splits of the dataset across the different folds has shown that presence of these outlier data instances in the test dataset (instead of in the train dataset) results in poor test accuracies. These outlier data instances have been found to mostly belong to specific Fe-based alloy compositions, which do not have any information corresponding to the alloy processing history in the “comments” and “heat treatment” columns. This explains the reason for poor model performance in case of these outlier points. It further confirms the importance of this information for accurate prediction of the pitting potential.

#### Composition optimization

**Fig. 3.**
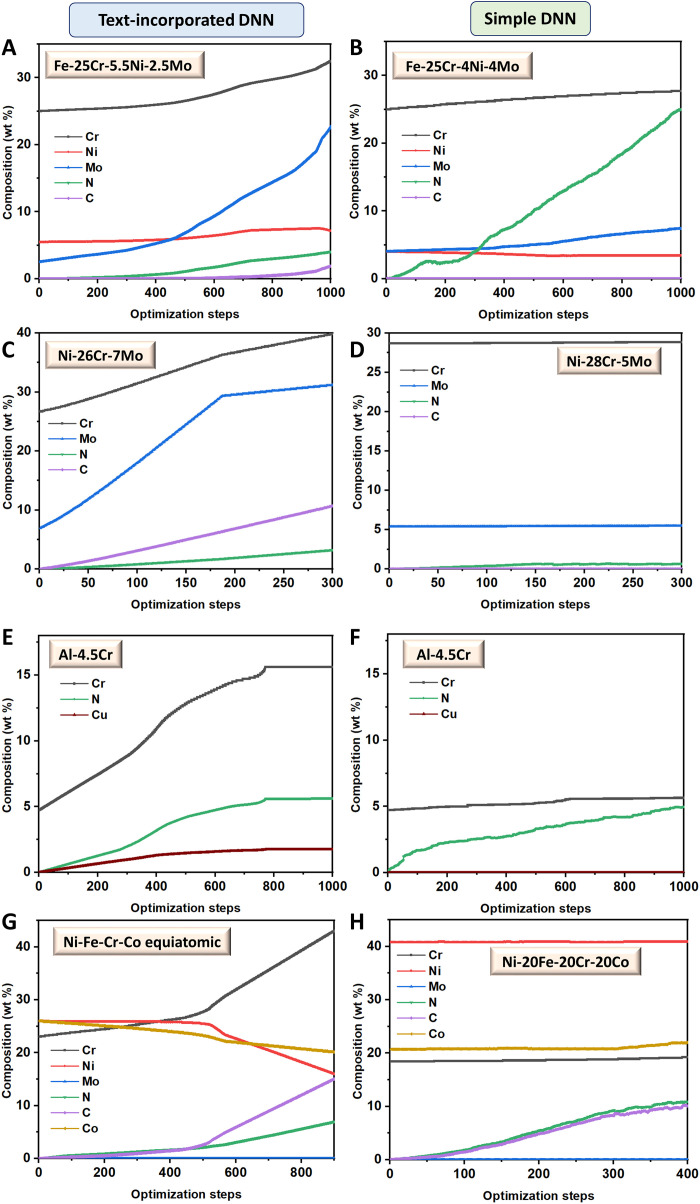
Composition optimization results. Comparison of compositional optimizations performed using the current process-aware DNN model [plots on the left column, i.e., (A), (C), (E), and (G)] and the previously published more simplistic DNN model (*31*) [plots on the right column, i.e., (B), (D), (F) and (H)]. Comparable initial alloy compositions have been chosen from (**A** and **B**) Fe-based alloys, (**C** and **D**) Ni-Cr-based alloys, (**E** and **F**) Al-based alloys, and (**G** and **H**) high-entropy alloys.

Composition optimizations have been carried out using the process-aware DNN model, in particular to understand the differences in training outcomes in comparison to the previously published simple DNN model (*31*) without textual information. For this purpose, composition optimizations starting from similar initial alloy compositions and using the same learning rate have been carried out with both the simple and the process-aware DNN model variants. Figure 3 shows representative results of these optimizations. In all cases, the pitting potential was attempted to be maximized while maintaining the test environment parameters (i.e., pH value and chloride ion concentration) constant during the course of optimization and allowing only the compositional parameters to change. Apart from qualitative similarities in the Fe-based stainless steel (Fig. 3, A and B) and the FeCrNiCo high entropy alloy (Fig. 3, G and H), notable differences in the predicted composition trajectories between the two models can be observed. The first one is the notably enhanced contribution of Mo toward enhancing the pitting potential in the ferritic stainless steel (Fig. 3A) and the Ni-Cr-Mo alloy (Fig. 3C). Second, interstitial N and C have emerged as important contributors also to the Ni-Cr-Mo alloy with the process-aware DNN (Fig. 3C). With the simple DNN model, the NiCrMo alloy is actually the singular exception where neither of N and C can be seen to create a major impact (Fig. 3D). Last, the emergence of Cu as a positive contributor to the pitting potential of the Al-Cr alloy with the process-aware model can be seen as the additional factor not observed in the optimizations from the simple DNN model (Fig. 3, E and F).

The observed differences show the enhanced ability of the current process-aware DNN model toward comprehending the alloying element contributions to pitting resistance in the different alloy systems. This enhanced capability originates naturally from the enhanced information density provided as input for the model. For instance, Cu is an element that is known to enhance the pitting resistance of Al alloys (although only when in solid solution) (*42*, *43*). Despite this fact, the reason why it does not show a major contribution in the simple DNN model optimizations can be understood in the following manner. The database reports a pitting potential of −360 mV for a binary Al–4 wt % Cu alloy when measured by slow scan rate potentiodynamic polarization experiments. However, the same composition at the same pH and comparable chloride ion concentration has also been reported to have a pitting potential close to −600 mV when measured by potentiostatic measurements. In other words, the simple DNN model, because of its inability to read and use any textual data, perceives an apparently contradictory behavior of the Al-Cu alloys. Thus, no conclusive understanding of the beneficial/detrimental influence of Cu in this alloy system can be learned. This could just be a representative example of several such instances in the dataset.

Consequently, results from the process-aware DNN model, reflecting the emergence of Cu in the Al-Cr alloy, show that the information supplied by the textual data (in the form of a test methodology in the example cited here) plays an important role toward resolving these apparent contradictions. This also explains the higher test accuracies achieved. Further, the tendency for saturation in the composition alteration during the optimization in case of the Al-Cr alloy is quite noteworthy. It is well known from the literature (*42*, *43*) that the presence of Cu in excess of its solubility limit, resulting in the presence of Cu-rich precipitates, is not beneficial. These precipitates are cathodic to the surrounding Cu-lean matrix, making the interfacial regions preferred sites for pit initiation (*43*). It is therefore very encouraging to see that despite not imposing any artificial limits on any alloying elements during composition optimization, the model saturates at a Cu content (Fig. 3E) close to its solubility limit in the alloy matrix. This successful prediction could be attributed to the fact that the database consists of Cu contents varying in the range of 0 to 6 wt %, i.e., from below to much beyond the solubility limit.

Similarly, the enhanced contribution of Mo in the stainless steel and the Ni-Cr-Mo alloy (Fig. 3, A and C) and that of interstitial elements N and C in the Ni-Cr-Mo alloy (Fig. 3C) in optimizations using the process-aware DNN is also encouraging. This is because, in case of stainless steels, the coefficient for Mo in the pitting resistance equivalent number (*44*) has a much greater contribution toward enhancing pitting resistance than that reflected from the optimizations using the simple DNN. Nevertheless, it must be admitted that the model still does not naturally predict a saturation in either Mo or interstitial contents during the optimizations beginning from the transition-metal alloy–based compositions. This leads to possible overshooting of the modeled alloying element contents (such as 20 to 30 wt % Mo in Fig. 3, A and C) during optimization. Such an overestimation of at least one element even by the process-aware model further underscores the importance of embellishing the amount of training data for achieving better and reasonably capped results. Any efforts in this direction can be expected to turn out to be highly beneficial.

#### Keyword analysis for text inputs

While the results described above demonstrate a general importance of considering textual input, we now describe a strategy for identifying specific keywords/phrases within a given textual input that play the most crucial role in pitting potential prediction. This involves a customized input perturbation approach, which is demonstrated with the help of simple examples. A list of distinct textual inputs is generated by sequentially adding one word each of a given complete sentence. For instance, the list of inputs generated for the sentence, “Polarization curves were obtained in potentiostatic manner” (which serves as a test method input for an Fe-15Cr-13Ni-2.5Mo alloy composition) is presented in Table 2. This list of textual inputs for the feature in question is supplied to the trained process-aware DNN model while keeping all other inputs fixed. The variation in the pitting potential predicted by the trained model over consecutive inputs would represent the sensitivity/importance of the newly added word to the preceding phrase. The results obtained from this sentence clearly reveal the word “potentiostatic” to be the most important keyword in the sentence. The magnitude of error drops sharply when the word potentiostatic is introduced. A feature that is important to note is also the substantial variation in predicted pitting potential just before the appearance of the word potentiostatic. This is because the LSTM layers have the capacity to detect structure of phrases/sentences, as described earlier. In this case, the training data contain a lot of phrases of the form, “were obtained/measured in potentiostatic/potentiodynamic manner,” which appears to be recognized by the model.

**Table 2.** List of text inputs and the corresponding predicted pitting potentials using the process aware DNN model for a Fe-15Cr-15Ni-2.5Mo alloy for the purpose of keyword analysis.

S. no.	Text input for the test method feature	Predicted pitting potential (mV)	Error (mV)
1	Polarization	198.93	−27.07
2	Polarization curves	198.55	−27.45
3	Polarization curves were	199.88	−26.12
4	Polarization curves were obtained	199.90	−26.10
5	Polarization curves were obtained in	177.53	−48.47
6	Polarization curves were obtained in potentiostatic	233.18	7.18
7	Polarization curves were obtained in potentiostatic manner	239.59	13.59

However, it must also be mentioned that a similar analysis has also revealed some example sentences, which do not show any variations. For instance, for the same input of the Fe-15Cr-13Ni-2.5Mo alloy, a similar word-by-word analysis of the heat treatment input “This one received a final heat treatment of 1h at 1049C followed by water quenching compared to the one above” showed no particular variation at any word. This demonstrates the caution that needs to be exercised in performing such a keyword analysis. The strongly supervised nature of the LSTM layer training implies that keywords could be highly context sensitive (i.e., dependent on other input values, other phrases in the same input) and that it is not necessary to always have “keywords,” but a sentence as a whole could be important.

### Feature transformed DNN

#### Model architecture

For preparing input data, the compositional parameters of the alloys have been transformed into a set of different descriptors making use of different atomic, physical, and chemical properties of the constituent elements. This has been done primarily by using a specific alloy-composition-featurization function called “WenAlloys” available from “matminer,” an open-source Python library (*45*) providing featurization tools for performing machine learning tasks on materials data sets. The descriptors generated by this featurization process (which are used as the material-specific input parameters for the DNN model) and their definitions are tabulated in Table 3.

**Table 3.** Set of descriptors calculated from individual alloy compositions, serving as input parameters for the feature-transformed DNN model. *c_i_*, *r_i_*, χ*_i_*, and *E*_*c*,*i*_ refer to the atomic fraction, atomic radius, Pauling electronegativity, and cohesive energy of an element *i* in a given alloy composition. $\text{Δ}H_{MO_{x}}^{0}$ is the ground state formation enthalpy of an elemental oxide MO*_x_*. *R* is the universal gas constant.

Descriptor	Definition
Yang radii δ	$\sqrt{\sum_{i=1}^{n}c_{i}{\left(1-r_{i}/\bar{r}\right)}^{2}}$
Omega	$\frac{T_{m}\text{Δ}S_{\mathrm{mix}}}{|\text{Δ}H_{\mathrm{mix}}|}$
APE mean	Radius ratio between the central atom and the average radius of atoms in the nearest-neighbor shell, normalized by the ideal radius ratio for a cluster with that number of atoms
Radii local mismatch	$\sum_{i=1}^{n}\sum_{j=1,j\neq i}^{n}c_{i}c_{j}|r_{i}-r_{j}|$
Radii γ	$\frac{\left(1-\sqrt{\frac{{\left(r+r_{\min}\right)}^{2}-r^{2}}{{\left(r+r_{\min}\right)}^{2}}}\right)}{\left(1-\sqrt{\frac{{\left(r+r_{\max}\right)}^{2}-r^{2}}{{\left(r+r_{\max}\right)}^{2}}}\right)}$
Configurational entropy	$-R\sum_{i=1}^{n}c_{i}ln(c_{i})$
Lambda entropy	$\frac{\text{Δ}S}{\delta^{2}}$
Electronegativity δ	$\sqrt{\sum_{i=1}^{n}c_{i}{\left(\chi -\chi_{i}\right)}^{2}}$
Electronegativity local mismatch	$\sum_{i=1}^{n}\sum_{j=1,j\neq i}^{n}c_{i}c_{j}|\chi_{i}-\chi_{j}|$
Valence electron concentration (VEC) mean	$\sum_{i=1}^{n}c_{i}({\mathrm{VEC}}_{i})$
Mixing enthalpy	$\sum_{i=1,j>i}^{n}4c_{i}c_{j}.\text{Δ}H_{ij}^{\mathrm{mix}}$
Mean cohesive energy	$\sum_{i=1}^{n}c_{i}(E_{c,i})$
Number of itinerant electrons (total, s, p, d, and f)	$\sum_{i=1}^{n}c_{i}.{\left(e/a\right)}_{i}$
Mean oxide enthalpy	$\sum_{i=1}^{n}c_{i}.{\left(\text{Δ}H_{MO_{x}}^{0}\right)}_{i}$

Most of the descriptors have been developed within the high entropy alloy (*46*, *47*) and metallic glass (*48*) communities, with the aim of capturing different physical and chemical characteristics of multicomponent solid solutions into such mathematical descriptors (*46*, *49*, *50*). In addition to these features generated from matminer, we have added one more feature, namely, mean oxide enthalpy (also defined in Table 3) to incorporate information on the oxide stability of different elements within a given alloy composition. Test method–related parameters including test solution pH, chloride ion concentration, and test temperature have also been incorporated into the input training data. Information on microstructural details and materials class has not been included in this model since such information is partly encoded within the solid solution descriptors. It must also be noted that the textual input has not been included in this model, to first evaluate its performance in comparison with the previously published simple DNN model (*31*). The transformed feature space is then fed as input into a simple, fully connected DNN. The network consists of three hidden layers with 128, 64, and 32 nodes, respectively, in conjunction with a ReLU activation function and dropout fraction of 0.5 at each layer, before reaching the final output, i.e., the pitting potential.

#### Training and validation

**Fig. 4.**
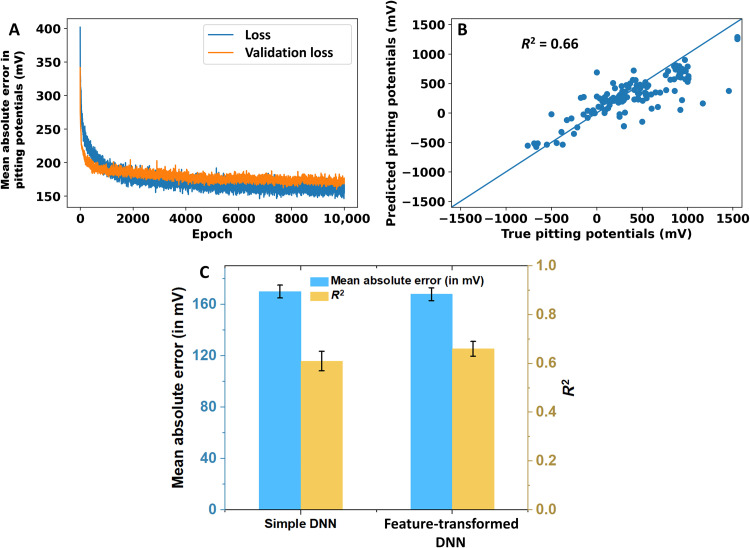
Model training results of the feature-transformed DNN. (**A**) Representative plot of the variation in the loss and validation loss measures, respectively, during training history (**B**) predictions of the trained model over the test dataset at the end of training. (**C**) Comparison of the model evaluation metrics of the simple and feature-transformed DNNs.

We now consider the second DNN model, which is taking the transformed features as input. For this model, a modification in the hyperparameters, i.e., increasing the number of nodes in the first hidden layer to 128, was essential. This was necessary for achieving similar training and test accuracies as in the published, simple DNN model (*31*). Figure 4 shows a representative variation in the error metrics (i.e., the loss and validation loss measures) during the 10,000 epochs of training. The validation loss and *R*^2^ coefficient for test predictions averaged over the sixfolds of cross-validation turned out to be 168 mV and 0.66, respectively (Fig. 4C). Overfitting has once again been avoided by virtue of using a dropout fraction of 0.5 at each hidden layer. The training and test accuracies were found to be rather similar during different folds of the sixfold cross-validation. Analysis of outliers did not reveal any specific compositions that had consistently high prediction errors. The training and test accuracies of this model are poorer than the process-aware DNN model discussed above. Despite this, the additional benefits offered by such a model are twofold. On the one hand, input feature space optimizations performed using it provide mechanistic insights into alloy corrosion phenomena. On the other hand, such a model allows making predictions for alloy systems that are not present in the training data. Both of these aspects are discussed in the following sections.

#### Mechanistic insights derived from input feature space optimizations

Maximization of the pitting potential by the gradient-descent method using the feature-transformed DNN model has been performed, starting from five different initial input instances. While the DNN model is not given any information of the alloy composition/alloy class at any stage of training or optimization, the identity of each input instance in terms of its actual composition and alloy class has been recorded during data preparation, for better analysis and interpretability. The five different initial inputs used for optimization have been chosen to represent one composition from each of the five alloy classes existing within the original/untransformed dataset.

**Fig. 5.**
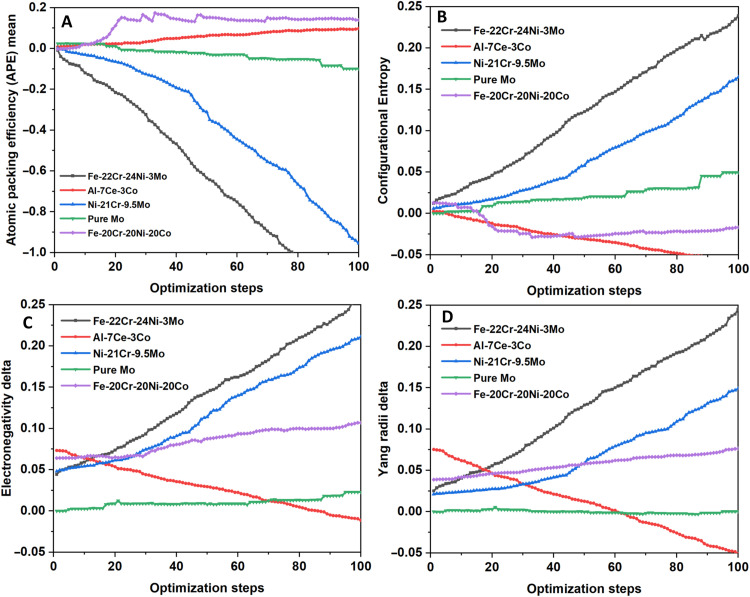
Optimization trajectories of the input features that exhibited rapid variation. (**A**) Atomic packing efficiency (APE) mean. (**B**) Configurational entropy. (**C**) Electronegativity δ. (**D**) Yang radii δ.

On the basis of the optimization trajectories, the input features can be classified into two categories: (a) those exhibiting rapid variation during optimization resulting in a substantial extrapolation beyond their respective ranges in the training dataset, and (b) those exhibiting negligible amount of variating during optimization. Figure 5 shows the trajectories followed by input features belonging to the first category, during the five optimization sequences. It can be inferred that these input features [i.e., “Yang radii δ,” “electronegativity δ,” “atomic packing efficiency (APE) mean,” and “configurational entropy”] would be the most important to be engineered, to achieve optimal pitting resistance in alloys.

Conventionally, two distinct, qualitative approaches that enhance the pitting resistance of an alloy have been discussed in the literature (*51*). One approach is to improve the stability and healing behavior of the protective, passive oxide film on the alloy surface. The other is to have alloying elements in the (surface regions of the) material that reduce the dissolution rate of the alloy in an active pit environment (*51*, *52*). The input features shown in Fig. 5 [i.e., those belonging to category (a)] can be viewed as quantitative descriptors that promote the occurrence of these conventionally recognized, qualitative pitting resistance mechanisms (in particular the latter), as elaborated below. It can be seen from Fig. 5 that the input feature trajectories starting from the initial instance corresponding to an Al-alloy composition are qualitatively opposed to those starting from other (transition-metal based) alloys. Therefore, a discussion of these transition-metal–based alloys is first presented, followed by a separate discussion on Al-based alloys.

First, a strong tendency to move toward negative values of the APE for the transition-metal–based alloys can be observed (Fig. 5A). APE is a quantity that has been designed within the metallic glasses community from a glass formability perspective (*48*, *53*). It represents the effective packing fraction of a solute-centered cluster of atoms independent of its configuration and applied strain. It is defined as the radius ratio between the central atom and the average radius of atoms in the nearest-neighbor shell, normalized by the ideal radius ratio for a cluster with that number of atoms, which has been established for binary systems in the literature (*53*). A value of APE equal to zero represents the ideal packing efficiency. Positive values represent clusters with excess free volume and negative values represent clusters packed even closer than the established ideal packing considering binary systems. The optimization pathways thus reveal a strong tendency to move toward increasing packing density in the metallic lattice for enhanced pitting resistance. An increased packing efficiency leads to an increase in the number of bonds to be broken for dissolving unit volume of the metal. In other words, high packing efficiency in the metallic lattice would necessitate a relatively low rate of metal dissolution in an active-pit environment and thus contribute to better pitting resistance. Similarly, increase in the local electronegativity differences between alloying elements would increase the strength of the metallic bonds, also making metal dissolution difficult. The increasing values of electronegativity δ for trajectories starting from transition-metal–based alloy parameters (Fig. 5C) are in corroboration with this understanding. Further, the corresponding configurational entropies can also be observed to be increasing for these trajectories initiating from the transition-metal–based alloy parameters (Fig. 5B). This is in support of the understanding that stabilization of single-phase solid solutions in preference to second-phase precipitation is generally good for pitting resistance. Last, the parameter related to atomic radii mismatch is also observed to have a strong influence on the pitting potential. In particular, the higher the mismatch, the higher is the pitting potential. While there appears to be no obvious reason for why an increased mismatch in radii should be beneficial for pitting resistance, such a correlation picked up by the DNN model could possibly be explained as follows. From the composition-based DNN models [in this work (Fig. 3) and also in the previously published work (*31*)], it can be seen that interstitial elements such as N and C have a strongly beneficial influence on the pitting potential. As these are elements of substantially different atomic radii than the other substitutional alloying elements in all alloy classes that constitute the main crystal lattice, an increase in their amounts obviously leads to an increase in the atomic radii mismatch. In other words, the strongly positive gradient in the Yang radii δ parameter could be interpreted as an indicator toward having substantial fractions of interstitial elements, i.e., elements with a distinctly smaller atomic radius than the host matrix element so as to improve pitting resistance.

The substantially different behavior of the optimization pathways in the region of the feature space corresponding to Al-alloys from the transition-metal–based alloy classes can be understood as follows. In case of Al alloys, the major contributor to the protective passive oxide film is the main matrix element, i.e., Al. Passive film formation is not dependent on alloying elements. Instead, the presence of alloying elements (such as Cu, Si, Mg, and Zn) leads to potential disruptions in the passive film formation owing to the presence of second-phase precipitates. The primary role of these alloying elements is toward improving the mechanical properties. In traditional alloy design, their amounts have been optimized in such a way that this mechanical property enhancement is achieved with minimal deterioration in the corrosion resistance. On the contrary, in other alloy classes such as stainless steels and Ni-based alloys, the main matrix elements, i.e., Fe or Ni, are not responsible for the formation of the protective oxide film. Instead, it is the presence of increasing amounts of Cr that results in its formation. Because of this fundamental difference in the mechanism of passivation, the behavior of Al-alloys differs notably from the rest of the alloy classes considered in this work. The trajectories of all the important input features indicate this deleterious influence of alloying in Al alloys in general. For example, the configurational entropy and the radii mismatch (Fig. 5, B and D) tend to decrease for maximizing the pitting potential, indicating a tendency to move toward the pure constituent Al. The tendencies for electronegativity mismatch and the atomic packing efficiency (Fig. 5, A and C) are also opposite to those in the other alloy classes, although with a lesser magnitude.

However, it must be stated that the magnitude of variation for all the input features in case of the optimization pathway beginning from the Al-alloy composition is much smaller than those beginning from other transition-metal–based alloy classes. This suggests a difficulty in identifying clear optimization pathways in the input feature space surrounding the Al-based alloys. This could be due to the much higher sensitivity of Al-alloys to processing history and test methodology, information that has not been provided to the current DNN model.

#### Prediction for Al-Cu-Sc-Zr alloy

As introduced earlier, one of the unique advantages that arises from training the DNN model on a transformed feature space is the ability to use the same trained network for making predictions on alloys containing elements that do not even exist within the training data. From the known elemental properties (such as atomic size, electronegativity, etc.), the input features can be calculated for any unseen alloy composition. If the DNN were to be trained on composition inputs only, then making such predictions would be impossible. We now demonstrate this capability of the DNN model by making predictions for a range of compositions in the Al-Cu-Sc-Zr alloy system. The training data used in the current work do not have any instances consisting of either Sc or Zr. Alloying of Al-Cu, Al-Mg, and Al-Cu-Mg-Zn alloys with Sc and Zr has been consistently observed to cause an enhancement in their corrosion properties, including pitting resistance (*54*–*57*).

**Fig. 6.**
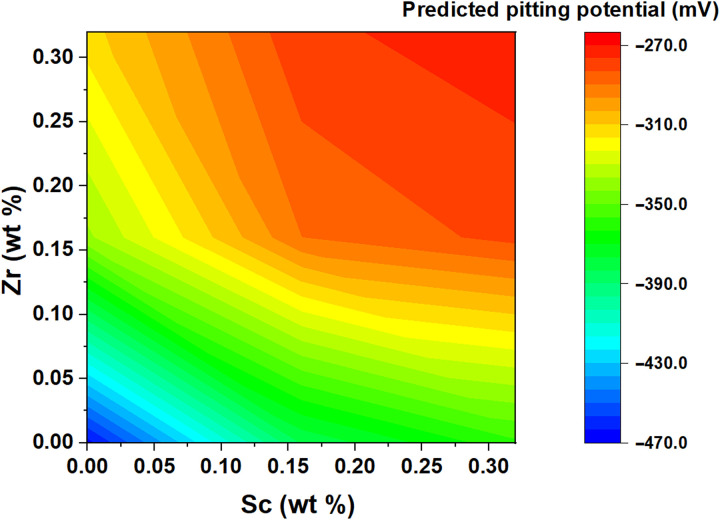
Predicted pitting potentials in the Al-Cu-Sc-Zr alloy system using the trained feature transformed DNN model. Each alloy composition is considered with a constant Cu content of 4 wt %.

Figure 6 shows a plot of the pitting potentials predicted for a series of Al–4 wt % Cu alloys, with Sc and Zr contents varying in the typical range of 0 to 0.3 wt %, respectively. For making the predictions, the alloy compositions were featurized by the same procedure followed for preparing the training data and provided as input to the trained DNN model. The beneficial influence of the presence of Sc and Zr can be seen with a steady increase in the pitting potential with increasing Sc and Zr contents. These results show the ability of the model to learn the effect of alloying elements, not only from correlations but also directly from their intrinsic physical and chemical properties, i.e., with causal background. Of course, the DNN model at this stage does not have any information of the effect of heat treatment, and therefore, the exact quantitative predictions may not be fully reflective of the experimental measurements. Nevertheless, by combining both methodologies described in this work, i.e., input feature transformation and natural language processing to describe processing history, we shall have a powerful tool at hand to make quantitative evaluations of uninvestigated alloy compositions. Such efforts, together with automated methods of data collection from the literature, in our opinion would be the way forward.

In summary, the process-aware neural network model capable of accepting textual inputs pertaining to alloy processing history and electrochemical test methodology in addition to numerical inputs was found to substantially outperform the simple neural network model in terms of pitting potential prediction accuracy. Alloying element contributions toward pitting resistance in a given electrochemical environment could be more accurately discerned using the process-aware neural network model in comparison to the simple neural network model. Positive contributions of Mo in stainless steels and Ni-based alloys, interstitial C and N in Ni-based alloys, and dissolved Cu in Al-based alloys were found to be noteworthy examples. Keyword analysis of the natural language processing–based process-aware model provided a methodology for identifying potential sources of crucial information present within the textual data. While the pitting potential prediction accuracy of the feature-transformed neural network model was found to be inferior in comparison to the process-aware neural network model, it provided valuable mechanistic insights into the alloy corrosion phenomenon. Alloy descriptors including configurational entropy, atomic packing efficiency, local electronegativity differences between alloying elements, and their atomic radii differences proved to be the most critical parameters providing pitting resistance. Through the example of the Al-Cu-Sc-Zr alloy system, we could also demonstrate the ability of the feature-transformed neural network model to make predictions on the role of alloying elements, Sc and Zr in this example, that might not be present in the training data.

## METHODS

### Model training

Both the models discussed in this work have been implemented, trained, and tested using Keras (*58*), an application programming interface written in Python, running on top of the machine learning platform TensorFlow (*59*). All the weights at the beginning of training are initialized in conjunction with the glorot uniform initializer (*60*) and the biases set to zero. The Adam optimization algorithm (*61*) was used during training with a step size (learning rate) of 0.001. For both models, the adapted datasets include 769 records. The respective datasets were divided into training and testing data sets in a 4:1 ratio, while ensuring random sampling in both. Also, in both cases, the models were trained six times, each time using a different random dataset split (i.e., sixfold cross-validation).

### Optimization

After training, optimization within the input parameter space of both the models was done to maximize the pitting potential using the multidimensional gradient descent algorithm (*62*), using a learning rate of 0.0001 in all cases. The augmented Keras model class, capable of returning the derivative of the output with respect to the inputs (AugNet) (*31*, *63*) has been used for this purpose with both the models. In case of the process-aware DNN, gradients were calculated only with respect to the numerical inputs. In other words, the text information contributes toward altering the weights of the different inputs within the trained model. However, they do not participate during the optimization process itself. In case of the feature-transformed DNN, gradients are calculated with respect to all the input parameters and maximization of the pitting potential is done while allowing the variation of all input parameters.
